# 1347. Comparison Between SARS-Cov-2, non-SARS-Cov-2 Coronavirus, Influenza and RSV Infections Among Solid Organ Transplant Recipients

**DOI:** 10.1093/ofid/ofab466.1539

**Published:** 2021-12-04

**Authors:** Maria A Mendoza, Motoa Gabriel, Mohammed Raja, Shweta Anjan, Anmary A Fernandez, Steve Courel, Aditya Chandorkar, Christopher O’Brien, Anita Phancao, Neeraj Sinha, Rodrigo Vianna, Ciancio Gaetano, Mathias Loebe, Jacques Simkins, Jose F Camargo, Michele I Morris, Lilian M Abbo, Giselle Guerra, Yoichiro Natori

**Affiliations:** 1 Jackson Memorial Hospital, MIAMI, Florida; 2 Jackson Memorial Hospital/ University of Miami, Miami, Florida; 3 University of Miami Miller School of Medicine/Sylvester Comprehensive Cancer Center, Miami, Florida; 4 University of Miami / Jackson Memorial Hospital, Miami, Florida; 5 Jackson Memorial Hospital/Miami Transplant Institute; University of Miami School of Medicine, Tampa, Florida; 6 University of Miami, Miami, Florida; 7 University of Minnesota, Minneapolis, Minnesota; 8 Jackson Memorial Hospital/ Miami Transplant Institute, Miami, Florida; 9 Jackson Memorial Hospital/ Miami Transplant Institute, University of Miami Miller School of Medicine, Miami, FL; 10 University of Miami Miller School of Medicine, Miami, FL; 11 University of Miami Miller School of Medicine & Jackson Health System, Miami, Florida; 12 Jackson Memorial Hospital/Miami Transplant Institute, University of Miami Miller School of Medicine, Miami, FL

## Abstract

**Background:**

Severe acute respiratory syndrome coronavirus 2 (SARS-CoV-2) pandemic has been raging since the end of 2019 and has shown worse outcomes in solid organ transplant recipients (SOTR). The clinical differences as well as outcomes between these respiratory viruses have not been well defined in SOTR.

**Methods:**

This is a retrospective cohort study of adult SOTR with nasopharyngeal swab or bronchoalveolar lavage PCR positive for either SARS-CoV-2, non-SARS-CoV-2 coronavirus, influenza, or respiratory syncytial virus (RSV) from January 2017 to October 2020; both inpatient and outpatient. The follow up period was up to three months. Clinical characteristics and outcomes were evaluated. Development of lower respiratory tract infection (LRTI) was defined as new pulmonary infiltrates with or without symptoms. For statistical analysis, Fischer’s exact test and log rank test were performed.

**Results:**

During study period, 157 SARS-CoV-2, 72 non-SARS-CoV-2 coronavirus, 100 influenza, 50 RSV infections were identified. Patient characteristics and outcomes are shown in tables 1 and 2, respectively. Secondary infections were not statistically significantly different between SARS-CoV-2 vs. non-SARS-CoV-2 coronavirus and influenza (p=0.25, 0.56) respectively, while it was statistically significant between SARS-CoV-2 and RSV (p=0.0009). Development of LRTI was higher in SARS-CoV-2 when compared to non-SARS-CoV-2 coronavirus (p=0.03), influenza (p=0.0001) and RSV (p=0.003). Admission to ICU was higher with SARS-CoV-2 compared to non-SARS-CoV-2 coronavirus (p=0.01), influenza (p=0.0001) and RSV (p=0.007). SARS-CoV-2 also had higher rates of mechanical ventilation when compared to non-SARS-CoV-2 coronavirus (p=0.01), influenza (p=0.01) and RSV (p=0.03). With time to event analysis, higher mortality with SARS-CoV-2 as compared to non-SARS-CoV-2 coronavirus, influenza, and RSV (p=0.01) was shown (Figure 1).

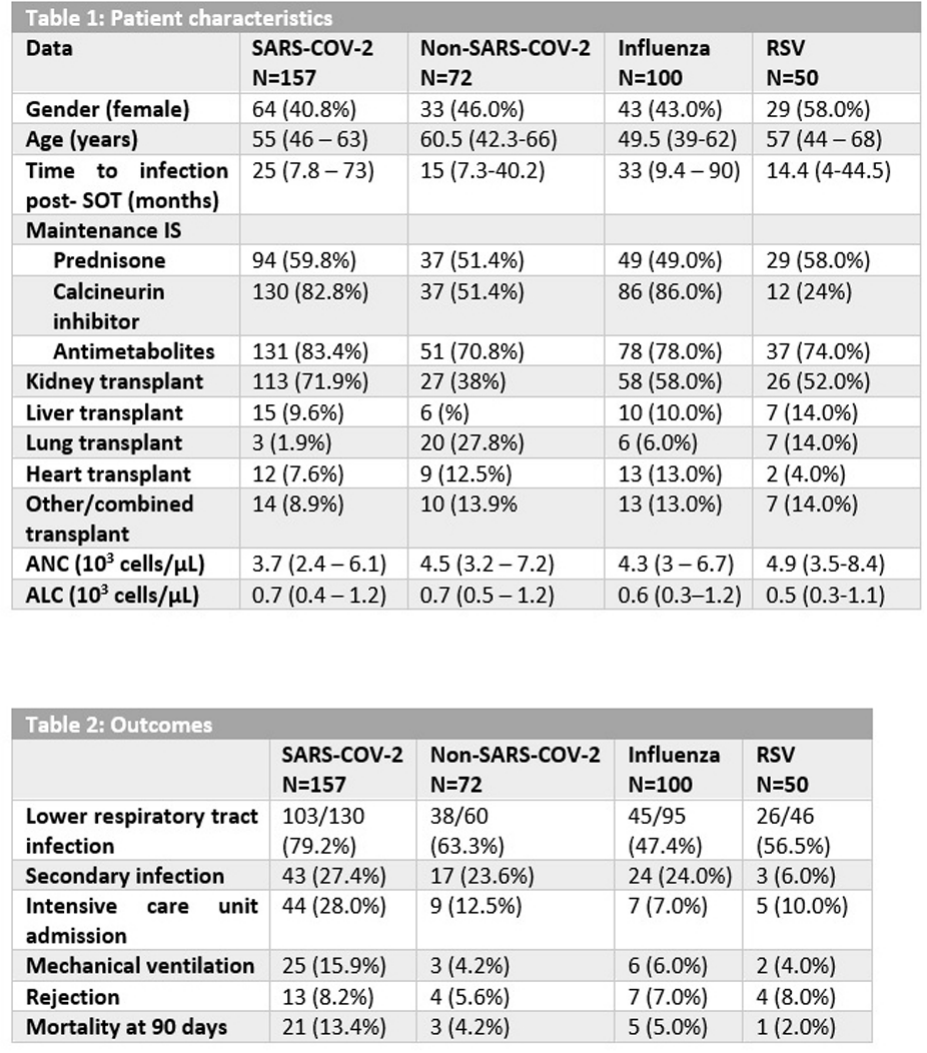

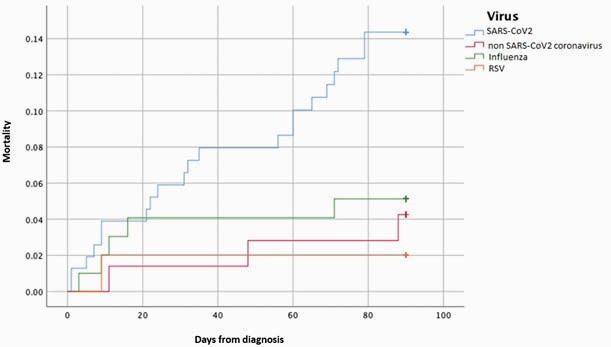

Figure 1. Kaplan Meier Curve: Comparison of Mortality between SARS-CoV-2, non-SARS-CoV-2 coronavirus, influenza and RSV

**Conclusion:**

We found higher incidence of ICU admission, mechanical ventilation, and mortality among SARS-CoV-2 SOTR vs other respiratory viruses. To validate these results, multicenter study is warranted.

**Disclosures:**

**All Authors**: No reported disclosures

